# Immunomodulatory activity of omadacycline *in vitro* and in a murine model of acute lung injury

**DOI:** 10.1128/msphere.00671-24

**Published:** 2024-10-30

**Authors:** Madeline Sanders, Paul Beringer

**Affiliations:** 1 Alfred E. Mann School of Pharmacy and Pharmaceutical Sciences, University of Southern California, Los Angeles, California, USA; University of Nebraska Medical Center College of Medicine, Omaha, Nebraska, USA

**Keywords:** acute lung injury, immunomodulatory, omadacycline, pharmacokinetics, pharmacodynamics

## Abstract

Cystic fibrosis (CF) is characterized by chronic airway obstruction, infection, and inflammation leading to progressive loss of lung function and eventual respiratory failure. Omadacycline, a tetracycline antibiotic, demonstrates *in vitro* activity against key CF pathogens, substantial lung penetration, and increasing clinical evidence for the treatment of lung infections in people with CF (PwCF). Preliminary *in vitro* data demonstrate that omadacycline exhibits anti-inflammatory activity. This study aims to determine the anti-inflammatory effects of omadacycline *in vitro* and in a murine model of lipopolysaccharide (LPS)-induced lung neutrophilia. *In vitro*, THP-1-derived macrophages were treated with omadacycline (20–100 µg/mL) 30 minutes prior to LPS stimulation. Pro-inflammatory cytokine (TNF-α, IL-1β/6), chemokine (CXCL-1/2), and MMP-9 levels were analyzed after 24 hours by ELISA. Omadacycline’s effects on IL-8-induced human neutrophil chemotaxis were also investigated. *In vivo*, omadacycline (2.5–30 mg/kg), comparators dexamethasone (1 mg/kg), and azithromycin (30 mg/kg) were administered 1 hour before and 6 hours after intranasal LPS challenge, respectively. Leukocyte counts and differentials in bronchoalveolar lavage fluid (BALF), inflammatory mediator levels in BALF and lung homogenates, pulmonary edema markers, and the severity of lung injury were evaluated 24 hours or 48 hours post-challenge. Treatment with omadacycline *in vitro* resulted in significant, dose-dependent reductions in IL-6, CXCL-1, and MMP-9 expression and inhibition of IL-8-induced neutrophil chemotaxis. *In vivo*, omadacycline yielded protective and therapeutic effects by reducing the production of proinflammatory cytokines and chemokines and neutrophil infiltration into the lungs, along with modestly improving lung injury severity. These preclinical results suggest that omadacycline may provide dual anti-bacterial and anti-inflammatory activities relevant to chronic lung infection treatment in PwCF.

**IMPORTANCE:**

Nontuberculous mycobacteria, particularly *Mycobacterium abscessus* complex (MABSC), are a major concern for people with cystic fibrosis (PwCF) due to their association with deteriorating lung function. A substantial barrier to effective treatment is the limited number of safe and effective antibiotics. Omadacycline offers a potential advancement in managing MABSC infections in cystic fibrosis due to its activity, effective penetration into pulmonary secretions, improved tolerability, and good oral bioavailability as shown in healthy volunteers. Our study is the first to explore omadacycline’s effects in a model of sterile lung inflammation and acute lung injury. We found that omadacycline not only has potent anti-bacterial properties but also exhibits anti-inflammatory effects, reducing lung inflammation and injury in our preclinical models. These findings underscore omadacycline’s potential as a dual-action therapy for lung infections in PwCF, indicating significant potential to improve patient outcomes and guide more effective antimicrobial therapy decisions.

## INTRODUCTION

Cystic fibrosis (CF) is an autosomal recessive, multisystemic disorder caused by mutations in the cystic fibrosis transmembrane conductance regulator (CFTR) gene. Over 1,900 known mutations can cause this disease, leading to dysfunction of the CFTR protein and disruption in ion and water transport across epithelial cells (1, 2). This causes increased mucus viscosity and defective mucus secretion, particularly in the respiratory and gastrointestinal systems. The thick mucus impairs mucociliary clearance and causes airway surface liquid depletion, leading to cycles of airway obstruction, inflammation, and infection. These complications contribute to bronchiectasis and the progressive loss of lung function (2). Despite significant therapeutic advances, including CFTR modulators, CF lung disease remains the leading cause of mortality in affected individuals (3).

The airways in CF are characterized by neutrophil-dominated inflammatory processes, predominately on the respiratory epithelial surface. Neutrophil-derived proteases [e.g., neutrophil elastase, matrix metalloproteinase 9 (MMP-9)] play a central role in the progressive destruction of airway tissues, disrupting elastin fibers and other matrix components (e.g., glycosaminoglycans and collagen), and are considered potential targets for therapeutic intervention (4). Elevated MMP-9 levels in the airways have been detected in bronchoalveolar lavage fluid (BALF) of people with CF (PwCF) and show a positive correlation with the severity of pulmonary disease (4, 5). CF-related airway inflammation is also associated with heightened production of pro-inflammatory cytokines such as tumor necrosis factor (TNF-α) and interleukin (IL)−1β, IL-6, and IL-8, which are closely linked to lung disease progression and reduced survival in PwCF (6). Moreover, chronic lung infections can exacerbate ongoing inflammatory lung damage by intensifying airway inflammation, leading to accelerated declines in lung function and reduced survival (7–9).

Currently, the number of safe and effective treatments for lung inflammation is limited. Oral corticosteroids and high-dose ibuprofen have demonstrated efficacy in reducing the rate of pulmonary function decline in children with CF. However, their use is restricted due to potentially significant side effects such as growth retardation, osteoporosis, and diabetes (with oral corticosteroids), as well as bleeding (with ibuprofen) (10, 11). Additionally, oral azithromycin has been shown to benefit PwCF who have chronic *Pseudomonas aeruginosa* infection, improving lung function and reducing the frequency of pulmonary exacerbations, though its efficacy appears to wane with time (12). Thus, there is a critical need for new anti-inflammatory agents. Specifically, agents with dual antibacterial and anti-inflammatory activities are increasingly attractive treatment options for PwCF who have or are at risk of developing chronic lung infections due to highly virulent organisms.

Tetracyclines as a pharmacologic class of antibiotics exhibit potent immunomodulatory activity and have shown efficacy in the treatment of a variety of inflammatory conditions including acne, periodontitis, rosacea, and rheumatoid arthritis (13). In experimental models of acute lung injury (ALI), tetracycline significantly diminished lung injury and pulmonary inflammation by selectively inhibiting caspase-1-dependent IL-1ß and IL-18 production, leading to improved survival rates. Tetracycline also reduced IL-1ß and IL-18 production by alveolar leukocytes in patients with direct acute respiratory distress syndrome (14). During acute pulmonary exacerbations in CF, oral doxycycline administration led to a significant 63.2% reduction in total sputum MMP-9 levels, along with improvements in forced expiratory volume in one second and a prolonged exacerbation-free interval (15).

Omadacycline is a novel third-generation tetracycline antibiotic that is currently approved for the treatment of community-acquired bacterial pneumonia (CABP) and acute bacterial skin and skin structure infections (ABSSSI) in adults (16). For CABP, omadacycline is administered as 200 mg intravenous (IV) dose on day 1, followed by 100 mg daily or 300 mg orally on day 1, and thereafter, while for ABSSSI, dosing starts with 200 mg IV or 450 mg orally, followed by 100 mg IV or 300 mg orally daily (16). Importantly, its pharmacokinetics have been thoroughly studied in healthy subjects, demonstrating that a 300 mg oral dose produces plasma exposures equivalent to a 100 mg IV dose and achieves effective pulmonary penetration (17, 18). Omadacycline also exhibits broad-spectrum activity against key pathogens, including *Mycobacterium abscessus*, methicillin-resistant *Staphylococcus aureus*, and *Stenotrophomonas maltophilia*, and has shown increasing clinical evidence supporting its efficacy in treating lung infections in PwCF (19–25). Recent studies, including two large multicenter longitudinal retrospective analyses (*n* = 117 and *n* = 75), support the long-term safety and tolerability of omadacycline for managing nontuberculous mycobacterial pulmonary infections (22, 23). Additionally, a separate case study of an adolescent with CF highlights its effective off-label use in combination therapies for treating *M. abscessus* (24). Preliminary *in vitro* data demonstrate that omadacycline exhibits anti-inflammatory activity (26). The effects of omadacycline in a model of sterile inflammation and direct lung injury, however, have not been previously investigated.

The potential dual anti-bacterial and anti-inflammatory properties of omadacycline offer a significant therapeutic advantage over current antibiotic therapies for managing both infection and inflammation in CF lung disease. To evaluate omadacycline’s immunomodulatory activity, we utilized a well-established murine model of intranasal lipopolysaccharide (LPS)-induced ALI, which mimics the neutrophil-driven inflammation observed in CF and other chronic lung diseases (27–29). In this model, omadacycline was administered to mice either 1 hour before or 6 hours after initiating pulmonary inflammation. We evaluated its effects *in vivo* by measuring airway neutrophil burden, levels of pro-inflammatory cytokines, chemokines, and MMP-9 release, as well as assessing pulmonary vascular leakage and the extent of lung injury. Additionally, *in vitro* assays examining neutrophil chemotaxis and LPS-induced macrophage inflammatory gene and protein expression provided supportive details on omadacycline-responsive cell populations. Our findings indicate that omadacycline reduces LPS-induced lung injury by inhibiting macrophage proinflammatory cytokine production and neutrophil recruitment.

## MATERIALS AND METHODS

### Measurement of LPS-induced cytokine/chemokine levels in THP-1-derived macrophages

#### Human cell cultures

The THP-1 cell line was purchased from ATCC and maintained at 37°C under 5% CO_2_ in RPMI 1640 medium (Corning) supplemented with 10% heat-inactivated fetal bovine serum (FBS; Genesee Scientific). THP-1 monocytes were differentiated into macrophages in the presence of 100 ng/mL phorbol-12-myristate 13-acetate (PMA; Sigma-Aldrich) for 48 hours, followed by 24 hours of rest in PMA-free culture media prior to starting experiments. For all experiments, THP-1 cells were used at passages 14–18.

#### Preparation of compounds

Bacterial LPS from *Escherichia coli* O111:B4 (Sigma-Aldrich) was dissolved in 1× phosphate-buffered saline (PBS; Cytiva) and subsequently diluted in culture medium. Omadacycline (Paratek Pharmaceuticals) and dexamethasone (MedChemExpress) were dissolved in dimethyl sulfoxide (DMSO; Sigma-Aldrich) and diluted in culture medium to a final DMSO concentration of 0.1% (vol/vol). The glucocorticoid dexamethasone was included as a comparator because of its known anti-inflammatory properties (30). For all *in vitro* assays, medium with 0.1% DMSO served as the vehicle control.

#### THP-1 cytotoxicity evaluation

THP-1 cells were seeded in triplicate at a density of 2 × 10^5^ cells/well into 96-well culture plates (Genesee Scientific) in RPMI 1640 complete medium plus 10% FBS and differentiated as described above. After a 30-minute pre-incubation at 37°C with either the vehicle or omadacycline (20, 40, 60, 80, or 100 µg/mL), THP-1-derived macrophages were stimulated with LPS (100 ng/mL) for an additional 24 hours. Incubation with the positive control substance Triton X-100 (2%) was used as a positive control of cell death. The media was aspirated, and the samples were gently washed twice with PBS. A 10% (vol/vol) alamarBlue solution was prepared by diluting the alamarBlue reagent (ThermoFisher Scientific) in culture media, and 200 µL of the 10% alamarBlue solution was added to each well. Background wells without cells were also included for background subtraction. Plates were incubated for 2 hours (37°C, 5% CO_2_), and their absorbance was measured at 570 nm and 600 nm as a reference, using a Sunrise microplate absorbance reader (Tecan). Cell viability, expressed as a percentage of vehicle-treated control cells, was determined by calculating the percentage reduction of the alamarBlue reagent based on absorbance readings, following the manufacturer’s instructions.

#### Cytokine expression analysis

THP-1 cells were seeded in triplicate at a density of 2 × 10^6^ cells/well into six-well culture plates (Genesee Scientific) in RPMI 1640 complete medium plus 10% FBS and differentiated as described above. THP-1-derived macrophages were then preincubated at 37°C with vehicle, dexamethasone (1 µM), or omadacycline (20, 40, 60, 80, or 100 µg/mL) for 30 minutes and subsequently stimulated with LPS (100 ng/mL) for an additional 24 hours. The cell supernatant was collected, aliquoted, and stored at −80°C. The frozen supernatant was thawed and thoroughly mixed, and concentrations of tumor necrosis factor (TNF)-α, IL-1β, IL-6, chemokine (C-X-C motif) ligand (CXCL)−1, CXCL-2, and MMP-9 were analyzed by ELISA (ThermoFisher Scientific) per the manufacturer’s protocol and using a Sunrise microplate absorbance reader (Tecan). Concentrations in samples were determined by interpolation from standard curves.

### Cytokine/chemokine mRNA expression levels in LPS-induced THP-1-derived macrophages

#### Cell processing and cDNA synthesis

To determine the effect of omadacycline on inflammatory gene expression, THP-1 cells were seeded, differentiated, treated with omadacycline, and stimulated with LPS as described above. After 4 hours, the cell supernatant was aspirated, and total RNA was isolated using RNAzol RT (Sigma-Aldrich) according to the manufacturer’s protocol. Total RNA was treated with the Turbo DNA-free kit (Invitrogen) to remove genomic DNA contamination and purified by RNA Clean & Concentrator (Zymo Research). The first-strand cDNA was reverse transcribed using iScript Reverse Transcription Supermix (Bio-Rad). All mRNA processing steps employed RNase-free solutions and sterile, disposable labware, with surfaces routinely decontaminated using RNaseZap (Invitrogen).

#### PCR primers

Primer sequences for human TNF-α, IL−1β, IL-6, CXCL−1, CXCL-2, MMP-9, and ubiquitin C (UBC) were designed using GeneBank database sequences from the National Center for Biotechnology Information (Table S1). To further avoid the contamination of genomic DNA, primers were designed to cross exon-exon junctions. Primers were synthesized by Integrated DNA Technologies.

#### Real-time quantitative PCR

Real-time quantitative qPCR was performed using the QuantStudio 12K Flex Real-Time PCR System (ThermoFisher Scientific) using 384-well plates with 10 µL of total reaction volume. The SsoAdvanced Universal SYBR Green Supermix (Bio-Rad) was used as the qPCR master mix. The thermal cycle profile was as follows: initial denaturation at 95°C for 2 minutes followed by 40 cycles of denaturation at 95°C for 15 seconds and annealing and elongation at 60° for 1 minute. Each reaction was run in technical triplicates. The delta-delta Ct method was employed to quantify gene expression (31). Relative expression levels of all genes were calculated relative to the housekeeping gene, UBC (32). Gene expression was scaled relative to the control, where untreated control samples were set at a value of 1. Statistics were performed on log2-transformed data.

### Measurement of IL-8–induced chemotaxis of primary human neutrophils

#### Neutrophil isolation and culture

Human blood collection was approved by the University of Southern California’s Institutional Review Board (HS-14–00294). All methods were carried out in accordance with relevant guidelines and regulations. Whole blood was collected from a single healthy volunteer by venipuncture into Vacutainer collection tubes containing the anticoagulant K2EDTA (BD Biosciences). The isolation of peripheral blood neutrophils was performed using the EasySep Direct Human Neutrophil Isolation Kit (STEMCELL Technologies). Following isolation, neutrophils were centrifuged (350 × *g*, 5 minutes), resuspended in phenol red-free RPMI 1640 (Gibco) supplemented with 1% (wt/vol) bovine serum albumin (BSA; Sigma-Aldrich), counted using a hemocytometer, and adjusted to a density of 1.25 × 10^7^ cells/mL. Neutrophil purity and viability were >95%, as assessed by differential counts and trypan blue dye exclusion, respectively. Neutrophils were then seeded in triplicate at a density of 1 × 10^6^ cells/well into a 96-well culture plate (Genesee Scientific) and pre-incubated (37°C, 5% CO_2_) with either the vehicle or omadacycline (100, 250, 500, 750, or 1,000 µg/mL). After 1.5 hours, these neutrophils were either used for cytotoxicity evaluation or introduced into the chemotaxis assay.

#### Neutrophil cytotoxicity evaluation

Following pretreatment with omadacycline, 50 µL of neutrophil cell suspension (5 × 10^5^ cells) was supplemented with RPMI 1640 (1% BSA) and alamarBlue reagent (ThermoFisher Scientific) to achieve a 10% (vol/vol) concentration of the alamarBlue solution. Incubation with Triton X-100 (2%) was used as a positive control of cell death. Background wells lacking cells were included for background subtraction. Plates were incubated for 4 hours (37°C, 5% CO_2_), and their absorbance was measured at 570 nm and 600 nm as a reference, using a Sunrise microplate absorbance reader (Tecan). Cell viability, expressed as a percentage of vehicle-treated control cells, was determined by calculating the percentage reduction of the alamarBlue reagent based on absorbance readings, following the manufacturer’s instructions.

#### Chemotaxis assay

Chemotaxis experiments were performed in 96-well HTS Transwell plates with 3 µm pores (Corning). The chemotactic factor IL-8 was used as the chemoattractant. The bottom well of the chemotaxis chamber was filled with 50 µL of IL-8 (20 ng/mL; PeproTech) or medium alone (negative control). The upper filter was then carefully placed on top of the plate and visually inspected to confirm the membrane’s contact with the fluid in each well’s bottom compartment and ensure no air bubbles were present between the filter and the media in the lower wells. Then, 25 µL of the omadacycline-pretreated neutrophils (2.5 × 10^5^ cells) was carefully pipetted directly onto the membrane, and the lid was put on the plate. The neutrophils were seeded in technical duplicates and allowed to migrate for 1.5 hours (37°C, 5% CO_2_).

At the end of the experimental period, the Transwell plate and membrane were centrifuged (350 × *g*, 1 minute) to dislodge any migrated cells adherent to the underside of the filter membrane. The filter membrane was then removed, and the number of migrated neutrophils was quantified with the CellTiter-Glo 2.0 assay (Promega) according to the manufacturer’s instructions. In brief, an equal volume (125 µL) of the CellTiter-Glo 2.0 Reagent was added to each well. The plate was shaken for 2 minutes to induce cell lysis and subsequently incubated at room temperature for 10 minutes to stabilize the luminescent signal. Luminescence was measured using the BioTek Synergy HTX plate reader (Agilent) equipped with the BioTek Gen5 Software and an integration time of 1 second. The percentage of maximal chemotaxis was calculated by establishing the migrated cell count in the presence of chemoattractant alone as 100% after subtraction for the mean number of cells undergoing random migration as previously described (33). The response of omadacycline-treated cells under chemoattractant was normalized to the untreated control.

### Comprehensive assessment of pulmonary inflammation, edema, and acute lung injury in murine lungs

#### Animals and husbandry

All animal experiments were reviewed and approved by the Institutional Animal Care and Use Committee at the University of Southern California (Protocol 21249). Male 6–8-week-old BALB/c mice (Charles River Laboratories) were housed under standard temperature (20°C–24°C) and humidity (60%–65%) laboratory conditions, with a 12:12-hour light-dark cycle. The animals were provided standard laboratory chow and water *ad libitum*.

#### Reagents

LPS from *E. coli* O111:B4 (Sigma-Aldrich) was dissolved in sterile 1× PBS (Cytiva). Omadacycline (Paratek Pharmaceuticals) was dissolved in DMSO (Sigma-Aldrich) and subsequently diluted in sterile PBS to final concentrations of 0.25, 0.75, 1.5, and 3 mg/mL [0.3% (vol/vol) DMSO]. Dexamethasone (MedChemExpress) was dissolved in DMSO and subsequently diluted in sterile PBS to a final concentration of 0.1 mg/mL [0.1% (vol/vol) DMSO]. Azithromycin (Selleck Chemicals) was dissolved in DMSO and diluted in sterile PBS to a final concentration of 6 mg/mL [50% (vol/vol) DMSO]. This formulation was essential due to azithromycin’s high insolubility in water alone. For all *in vivo* assays, PBS with 0.1% DMSO served as the vehicle control.

#### Experimental procedures

Several experiments were performed in groups of mice to assess the pharmacokinetics, efficacy, and safety of omadacycline in a murine model of LPS-induced ALI. Acute neutrophilic airway inflammation and lung injury were induced in mice by intranasal instillation of bacterial LPS as previously described (27–29). Mice were sedated with isoflurane (VetOne) delivered via a vaporization and anesthesia device. Acute lung inflammation was induced by intranasal administration of *E. coli* O111:B4 LPS. The solution of LPS was prepared at 3 µg per µL of saline, and 1 µL/g of body weight was introduced (e.g., 25 µL for a mouse of 25 g) with a siliconized pipette tip during the inspiration. Efficacy was then determined in two dose-ranging studies for the prevention and treatment of ALI. No mortality was observed in the disease model.

In the dose-ranging prevention study, mice were randomly assigned saline [subcutaneous (SC)], dexamethasone [1 mg/kg; intraperitoneal (IP)], azithromycin (30 mg/kg; SC), or omadacycline (2.5, 7.5, 15, or 30 mg/kg; SC) 1 hour prior to LPS challenge (*n* = 6/group). The omadacycline doses and administration route were selected based on prior published data (34). The antibiotic azithromycin and the glucocorticoid dexamethasone were chosen as comparators due to their established anti-inflammatory properties. Their doses and administration routes were based on a prior published study and produced reproducible effects in the LPS-induced neutrophilia model (35). Twenty-four hours after LPS administration, mice were euthanized by intraperitoneal injection of a lethal overdose of Euthasol (Virbac).

In the dose-ranging treatment study, mice were randomly assigned saline (SC), dexamethasone (1 mg/kg; IP), azithromycin (30 mg/kg; SC), or omadacycline (15 or 30 mg/kg; SC) 6 hours after LPS challenge (*n* = 6/group). Forty-eight hours after LPS administration, mice were euthanized by intraperitoneal injection of an overdose of Euthasol (Virbac).

#### Terminal blood collection

Immediately following euthanasia, blood was collected via intracardiac puncture in EDTA-coated tubes. Plasma was promptly obtained through consecutive centrifugation steps at 1,200 g, 4°C for 10 minutes, followed by a second centrifugation at 2,000 rpm, 4°C for another 10 minutes, and subsequently stored at −80°C until analysis.

#### Bronchoalveolar lavage fluid collection and cytological analysis

Bronchoalveolar lavage was collected by flushing the lungs three times with 1 mL of cold PBS (Cytiva) without Ca^2+^/Mg^2+^. Collected BALF samples were immediately placed on ice, followed by clarification through centrifugation (400 × *g*, 10 minutes, 4°C). BALF supernatant was aliquoted and stored at −80°C. The resulting cell pellets were then resuspended in 250 µL PBS. Airway total white blood cell (WBC) counts were determined for each BALF sample using Turk blood diluting fluid (Ricca Chemical) and a hemocytometer. Approximately 100 µL of cell suspension was added to a slide chamber, spun at 700 rpm for 10 minutes in a Shandon Cytospin 3 cytocentrifuge (ThermoFisher Scientific), and stained using the Differential Quik III Stain Kit (Polysciences) according to the manufacturer’s instructions. Differential cell counts were performed with a light microscope and 40× objective lens for polymorphonuclear neutrophils and macrophages. Neutrophil-to-macrophage ratios were calculated using a deep learning AI (Biodock.AI) and averaged from five random fields per sample.

#### Lung tissue sampling

After bronchoalveolar lavage, whole mouse lungs were removed, separated into left and right lobes, snap-frozen in liquid nitrogen, and stored at −80°C until further analysis. Frozen lungs were thawed and homogenized in T-PER Tissue Protein Extraction Reagent (ThermoFisher Scientific) supplemented with Complete Mini protease inhibitor tablets (Roche). Samples were then mechanically homogenized using a Tissue-Tearor (Polytron). Blades of the homogenizer were sterilized with 10% bleach and subsequently rinsed with ethanol and PBS before beginning and after processing each sample.

#### LC-MS/MS analysis of omadacycline

Omadacycline concentrations in mouse plasma and BALF were determined using liquid chromatography-tandem mass spectrometry (LC-MS/MS) (Institute of Clinical Pharmacodynamics, Schenectady, NY). For plasma, the LC-MS/MS method utilized was linear (1/*x*^2^) over the concentration range 0.020–5.000 mg/L and the correlation coefficient (*r*^2^) values ranged from 0.9995 to 0.9996. For the method quality control plasma samples, the inter-assay coefficient of variance (%CV) ranged from 2.48% to 4.64%, and the inter-assay percent recovery ranged from 99.6% to 110.6%. For BALF, the LC-MS/MS method utilized was linear (1/*x*^2^) over the concentration range 0.001–0.250 mg/L, and the correlation coefficient (*r*^2^) values ranged from 0.9993 to 0.9997. For the method quality control plasma samples, the inter-assay coefficient of variance (%CV) ranged from 0.97% to 9.23%, and the inter-assay percent recovery ranged from 99.0% to 103.7%.

#### Pharmacokinetic studies in mice

Pharmacokinetics were performed in healthy mice receiving a single subcutaneous dose of 2.5, 7.5, 15, or 30 mg/kg omadacycline. Plasma and BALF samples were collected at the terminal time points of 0.25, 0.5, 1, 2, 6, 12, and 24 hours (*n* = 3/group). Plasma and BALF time-concentration profiles were analyzed by noncompartmental pharmacokinetic analysis using a sparse sampling and Bailer-Satterthwaite approach in WinNonlin Phoenix version 8.4 (Certara Inc.). Linear regression analysis was conducted using the maximum plasma and BALF concentrations (C_max_) and total drug exposure (AUC_0-24_) to assess dose linearity and proportionality.

#### Measurement of total protein and cytokines levels in BALF

The frozen BALF supernatant was thawed and thoroughly mixed, and the total protein concentration for each sample was determined using a BCA protein assay (Pierce, ThermoFisher Scientific). Concentrations of TNF-α, IL-1β, IL-6, CXCL-1, CXCL-2, and MMP-9 were analyzed by ELISA (ThermoFisher Scientific) per the manufacturer’s protocol and using a Sunrise microplate absorbance reader (Tecan). Concentrations in samples were determined by interpolation from standard curves.

#### Urea correction

Epithelial lining fluid (ELF) concentrations (C_ELF_) were determined by utilizing the urea correction methodology according to the following formula: C_ELF_ = C_BAL_ × [(urea)_plasma_/(urea)_BAL_]. In this equation, C_BAL_ is the concentration of protein measured in the BALF, (urea)_plasma_ is the concentration of urea in plasma, and (urea)_BAL_ is the concentration of urea in the BALF (36). The urea concentrations in plasma and BALF samples collected simultaneously at the time of bronchoscopy were analyzed by QuantiChrom Urea Assay Kit (BioAssay Systems) according to the manufacturer’s instructions and using a Sunrise microplate absorbance reader (Tecan). Concentrations in samples were determined by interpolation from standard curves.

#### Measurement of total protein and cytokines levels in lung tissue

After homogenization, concentrations of TNF-α, IL-1β, IL-6, CXCL-1, CXCL-2, and MMP-9 from lung homogenate supernatant were analyzed by ELISA (ThermoFisher Scientific) per the manufacturer’s protocol and using a Sunrise microplate absorbance reader (Tecan). Concentrations in samples were determined by interpolation from standard curves. The measured concentrations were normalized to total protein content, as determined by BCA protein assay (Pierce, ThermoFisher Scientific).

#### Measurement of lung wet/dry ratio

In separate studies, the severity of pulmonary edema was assessed by the wet-to-dry ratio (W/D). For the prevention and treatment studies, mice were treated as previously described (*n* = 3/group). Immediately after euthanasia, the lung wet weight for each mouse was recorded. The lungs were then incubated in an oven (80°C for 24 hours) to obtain a dry weight. The W/D ratio was calculated using the wet and dry weights.

#### Lung histopathology

In separate studies, the effects of omadacycline on the severity of LPS-induced acute lung injury were investigated. Mice were treated as previously described for both the prevention and treatment studies (*n* = 3/group). Immediately after euthanasia, the lungs were transcardially perfused with ice-cold PBS containing 10 U/mL of heparin to remove blood. They were then infused with 10% formalin-buffered solution (Sigma-Aldrich) until fully inflated (37). Subsequently, the lungs were resected, placed into cassettes, and fixed in 10% formalin-buffered solution for 24–48 hours, then dehydrated by gradient ethanol, embedded in paraffin, and sliced into 3 um sections. Tissue processing, embedding, and sectioning were conducted by the USC Mann Histology Laboratory. The slides were stained with hematoxylin and eosin (H&E) by the USC Norris Comprehensive Cancer Center Translational Pathology Core and scanned at 40× magnification with a Hamamatsu NanoZoomer S60 digital whole slide scanner. Analysis software NDP.view2 was used for image processing (Hamamatsu Photonics). Lung injury scores were evaluated by a pathologist who was blinded to the identity of the slides following a previously described lung injury scoring system (38).

#### Data and statistical analysis

Statistical and graphical analyses were performed using GraphPad Prism version 10.1 (GraphPad Software, Inc.). Data normality was evaluated through visual inspection, Q-Q plots, histograms, and by measuring skewness and kurtosis. Non-normal data were then log-transformed and re-evaluated using the same methods. Statistical differences between treatment groups were determined by one-way ANOVA with Tukey’s multiple comparison post-hoc tests. The strength and direction of associations were determined using Pearson’s correlation coefficient. A significance level of *P* less than 0.05 was determined *a priori*.

## RESULTS

### Omadacycline inhibits IL-8-Induced neutrophil chemotaxis

**Fig 1:**
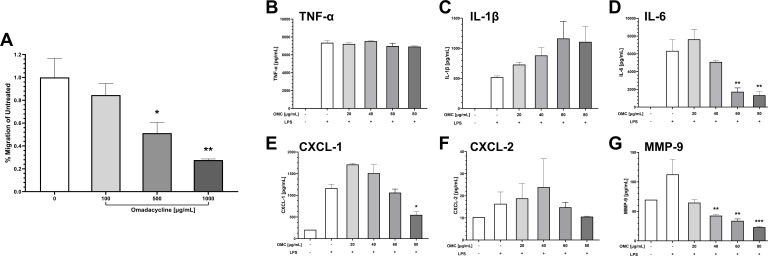
(**A**) Effects of omadacycline on IL-8-induced chemotaxis of primary human neutrophils. The degree of inhibition is expressed as the percentage of maximal chemotaxis. (**B–G**) Effects of omadacycline on cytokines and chemokines from LPS-stimulated THP-1-derived macrophages. Cell supernatant was evaluated for (**B**) TNF-α, (**C**) IL-1β, (**D**) IL-6, (**E**) CXCL-1, (**F**) CXCL-2, and (**G**) MMP-9. Data are represented as mean and SEMs. Asterisks indicate significant differences from vehicle-treated control cells. (*P < 0.05, **P < 0.01, ***P < 0.001, and ****P < 0.0001, post-hoc Tukey’s HSD test). OMC = omadacycline.

Given the critical role of airway neutrophilia in ALI pathogenesis, the activity of omadacycline was initially evaluated using *in vitro* methods. First, the effects of omadacycline on IL-8-induced chemotaxis of primary human neutrophils were evaluated using a standard Transwell migration assay. Pretreatment with omadacycline resulted in significant inhibition of neutrophil chemotaxis in a dose-dependent manner compared with control, with approximately 50% inhibition at 500 µg/mL (Fig. 1A). No statistically significant decreases in neutrophil viability were observed following omadacycline treatment (Fig. S1A).

### Omadacycline dampens LPS-induced inflammatory responses in THP-1-derived macrophages

Additional *in vitro* experiments were conducted using LPS-stimulated THP-1-derived macrophages to further assess omadacycline’s anti-inflammatory properties. Cell culture supernatant was utilized to analyze the expression profiles of relevant pro-inflammatory cytokines (TNF-α, IL-1β, and IL-6), chemokines (CXCL-1/2), and MMP-9 production (Fig. 1B through G). Concentrations of 40, 60, and 80 µg/mL of omadacycline led to significant reductions in MMP-9 levels (*P* < 0.01, *P* < 0.01, and *P* < 0.001, respectively; Fig. 1G). Significant decreases in IL-6 levels were also observed following treatment of 60 and 80 µg/mL omadacycline (*P* < 0.01; Fig. 1D). Similarly, CXCL-1 levels were markedly reduced with omadacycline treatment at 80 µg/mL (*P* < 0.05; Fig. 1E). There were no significant differences in the levels of TNF-α, IL-1β, or CXCL-2 observed with omadacycline treatment (Fig. 1B, C and F). Omadacycline treatment at 100 µg/mL was also tested but resulted in a statistically significant reduction in THP-1-derived macrophage cell viability (*P* < 0.05; Fig. S1B).

To investigate whether these actions could be explained by omadacycline-regulated gene expression, real-time qPCR was conducted to ascertain the mRNA expression levels of the target genes. The mRNA expression profiles were similar to the data generated from ELISA, with a moderately strong and significant positive correlation between gene and protein expression (*r* = 0.63; *P* < 0.001). CXCL-1 mRNA expression was downregulated for all doses of omadacycline treatment. Likewise, IL-6 and MMP-9 mRNA expression were attenuated with higher concentrations of 60, 80, and 100 µg/mL omadacycline. Interestingly, TNF-α transcription appeared to be upregulated for all doses of omadacycline treatment. No significant differences in mRNA expression were observed for IL-1β or CXCL-2 (Fig. S2).

### Pharmacokinetics of omadacycline in mice

**Fig 2:**
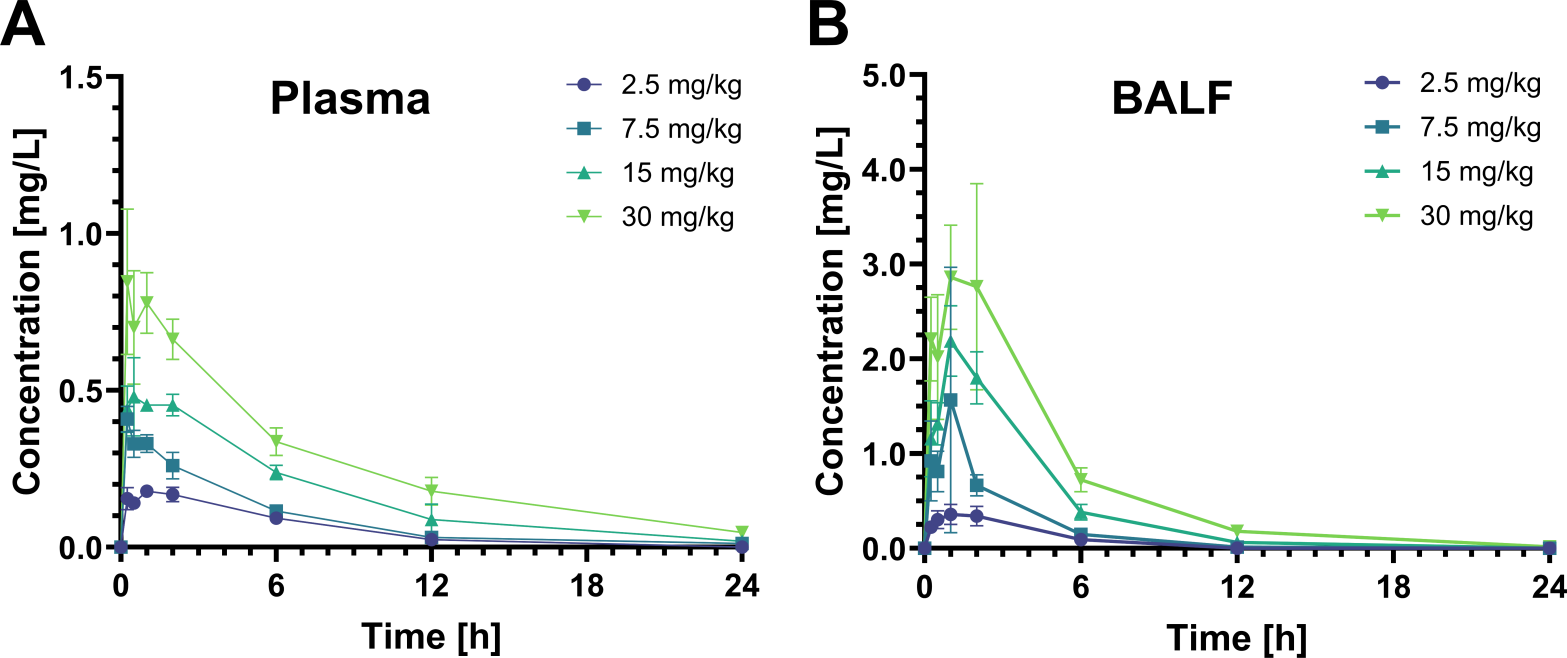
Plasma and BALF omadacycline concentration vs time profiles. (**A**) Omadacycline disposition in plasma after a single subcutaneous injection at 2.5, 7.5, 15, and 30 mg/kg. (**B**) Omadacycline disposition in BALF after a single subcutaneous injection at 2.5, 7.5, 15, and 30 mg/kg. Data are represented as mean and SEMs. *N* = 3 per group.

The pharmacokinetics of a single SC injection of omadacycline doses at 2.5, 7.5, 15, or 30 mg/kg were assessed in 84 healthy BALB/c mice (6–8 weeks old; mean weight, 21.0 ± 1.6 g). The mean plasma concentration vs time and mean BALF concentration vs time plots are depicted (Fig. 2). The total plasma and BALF observed maximum concentrations (C_max_), area under the concentration curve from dose time to the final observation time (AUC_0-24_), time to maximum concentration (T_max_), and half-life (t_1/2_) of omadacycline are reported (Table 1). In terms of distribution from plasma to BALF in mice, omadacycline showed rapid penetration into the lung compartment following a single SC dose, with mean maximum concentrations observed within 1 hour in the BALF. Its disposition appears to be biphasic with a short distribution phase followed by a prolonged elimination phase in plasma and BALF (mean, 5.2 and 2.9 hours, respectively). Observed C_max_ and AUC_0-24_ values exhibited linear dose proportionality. Across the omadacycline doses, the mean AUC_0–24_ ratio for BALF to plasma was approximately twofold (Table 1).

**TABLE 1:** Noncompartmental pharmacokinetic parameter estimates^*a*^

Parameter	Omadacycline (mg/kg)							
	2.5		7.5		15		30	
	Plasma	BALF	Plasma	BALF	Plasma	BALF	Plasma	BALF
*C*_max_ (mg/L)	0.178 (0.006)	0.357 (0.062)	0.408 (0.023)	1.565 (0.810)	0.480 (0.072)	2.188 (0.215)	0.846 (0.134)	2.861 (0.318)
AUC_0–24_ (mg*h/L)	1.182 (0.058)	1.470 (0.157)	2.123 (0.086)	4.238 (0.638)	3.889 (0.271)	9.021 (0.499)	6.288 (0.285)	15.864 (1.655)
*T*_max_ (h)	1	1	0.25	1	0.5	1	0.25	1
*t*_1/2, λz_ (h)	3.555	2.112	5.637	2.547	5.201	2.075	6.331	4.668

^*a*^ Mean values for each group are reported (*N* = 3/group). SEMs are in parentheses. C_max_ = observed maximum concentration, AUC_0–24_ = area under the mean concentration curve from dose time to the final observation time, T_max_ = time to maximum concentration, and *t*_1/2, λz_ = terminal elimination half-life.

### Omadacycline attenuates LPS-induced lung neutrophilia in mice

**Fig 3:**
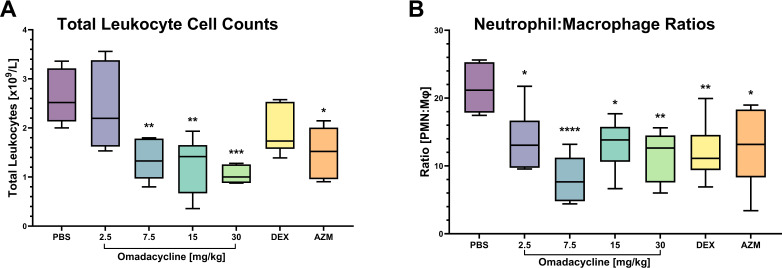
(**A**) Total leukocyte cell counts and (**B**) neutrophil to macrophage ratios in BALF after treatment of omadacycline and controls 1 hour prior to LPS challenge. Box and whisker plots show 25% percentile, median, and 75% percentile in box, with minimum and maximum values shown with whiskers. Asterisks indicate significant differences from untreated control animals (*P < 0.05, **P < 0.01, ***P < 0.001, and ****P < 0.0001, post-hoc Tukey’s HSD test.). PMN = polymorphonuclear neutrophil, Mφ= macrophage, DEX = dexamethasone, and AZM = azithromycin.

In the dose-ranging ALI prevention study, administering omadacycline treatment at doses of 2.5, 7.5, 15, and 30 mg/kg SC 1 hour prior to intranasal LPS challenge revealed a dose-dependent reduction in total WBC count and neutrophil recruitment to the lungs, observed in BAL measurements 24 hours post-challenge compared to the control group treated with PBS only (Fig. 3A and B). Total WBC counts were significantly reduced at omadacycline doses of 7.5 mg/kg (*P* < 0.01), 15 mg/kg (*P* < 0.01), and 30 mg/kg (*P* < 0.001) compared with PBS control (Fig. 3A). Neutrophil-to-macrophage ratios were significantly reduced at all omadacycline doses tested compared with PBS control (*P* < 0.05, *P* < 0.0001, *P* < 0.05, and *P* < 0.01, respectively; Fig. 3B). These reductions were comparable to or more potent than dexamethasone (1 mg/kg IP) and azithromycin (30 mg/kg SC), with a tendency toward more potent inhibition of total cell counts and neutrophil recruitment at the 7.5 mg/kg omadacycline dose. Similar effects were noted in animals in the dose-ranging ALI treatment study that received omadacycline treatment post-LPS insufflation (Fig. S3).

### Omadacycline inhibits LPS-induced pro-inflammatory cytokine, chemokine, and MMP-9 production in mice

#### Lung homogenate analysis

**Fig 4:**
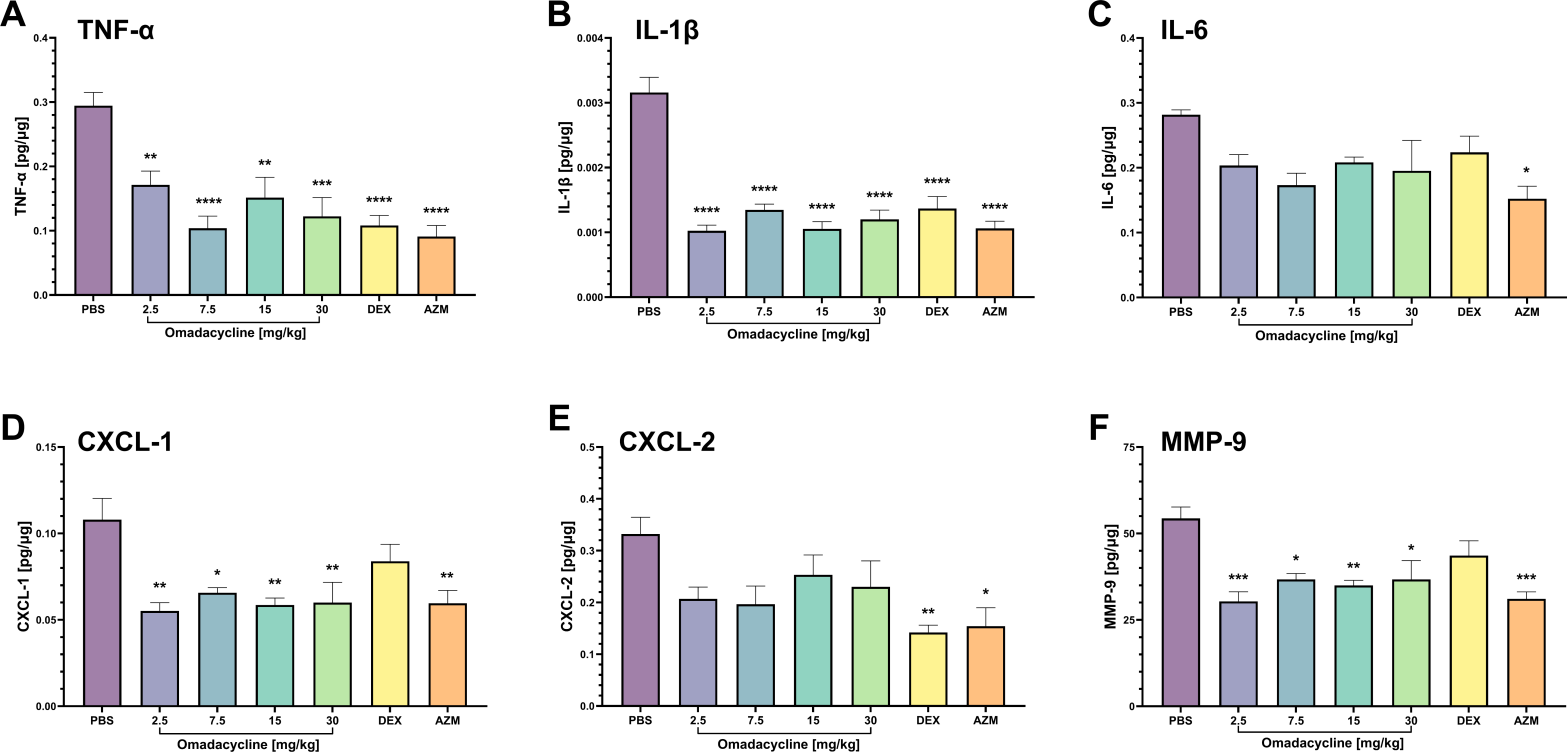
Dose effects of preventative omadacycline on lung homogenate cytokines and chemokines. Lung homogenate supernatant was evaluated for (**A**) TNF-α, (**B**) IL-1β, (**C**) IL-6, (**D**) CXCL-1, (**E**) CXCL-2, and (**F**) MMP-9. Data are represented as mean and SEMs. Asterisks indicate significant differences from untreated control animals (*P < 0.05, **P < 0.01, ***P < 0.001, and ****P < 0.0001, post-hoc Tukey’s HSD test). DEX = dexamethasone and AZM = azithromycin.

To evaluate the preventative effects of omadacycline on LPS-induced pro-inflammatory cytokines, chemokines, and MMP-9, ELISA was performed on mouse lung homogenate (Fig. 4A through F). TNF-α, IL-1β, CXCL-1, and MMP-9 concentrations were significantly reduced at all omadacycline doses tested compared with PBS control (Fig. 4A, B, D and F). Although there were no significant differences in the levels of IL-6 or CXCL-2 observed following omadacycline treatment, there was a noticeable trend toward reduced levels (Fig. 4C and E). Overall, the reductions seen with all tested omadacycline doses were similar to those observed with dexamethasone (1 mg/kg IP) and azithromycin (30 mg/kg SC). Additionally, significant inhibition of TNF-α, IL-6, and CXCL-2 was evident in the dose-ranging ALI treatment study with 15 mg/kg omadacycline administered 6 hours post-LPS challenge (Fig. S4).

#### BALF analysis

Anti-inflammatory effects were also assessed in BALF samples using ELISA and were normalized by the urea method as described above (Fig. S5A through F). Although no statistically significant differences were found, there was a strong positive association between lung homogenate and BALF concentrations (*r* = 0.97; *P* < 0.0001). Specifically, strong correlations were identified for TNF-α (*r* = 0.822) and CXCL-2 (*r* = 0.747).

TNF-α concentrations were reduced across all omadacycline doses (Fig. S5A), and dose-dependent decreases in IL-1β and CXCL-2 concentrations were also observed (Fig. S5B and E). Additionally, CXCL-1 concentrations exhibited a noticeable decrease at the highest omadacycline dose of 30 mg/kg (Fig. S5D). Generally, the reductions in these levels observed with the 30 mg/kg omadacycline dose were comparable to those observed with dexamethasone (1 mg/kg IP) and azithromycin (30 mg/kg SC). No treatment differences were observed for IL-6 or MMP-9 concentrations (Fig. S5C and F).

Similar anti-inflammatory effects were evident in the dose-ranging ALI treatment study. Once again, there was a strong positive association between lung homogenate and BALF concentrations (*r* = 0.95; *P* < 0.0001). Notably, TNF-α (*r* = 0.755), IL-6 (*r* = 0.804), and CXCL-2 (*r* = 0.985) levels showed strong correlations. Overall, treatment with 15 mg/kg omadacycline resulted in substantial reductions in the concentrations of TNF-α, IL-1β, IL-6, CXCL-1, CXCL2, and MMP-9 in BALF (Fig. S6).

### Omadacycline’s effect on pulmonary edema

Omadacycline’s potential to alleviate pulmonary edema *in vivo* was investigated by assessing total protein content in BALF, which serves as an indicator of alveolar permeability and the existence of pulmonary edema, alongside evaluating the severity of edema via lung wet-to-dry ratios. Treatment with omadacycline, dexamethasone, or azithromycin 1 hour prior to LPS challenge did not result in any statistically significant changes to total protein concentrations in BALF or lung wet-to-dry relationships in the murine model of LPS-induced lung neutrophilia (Fig. S7A and B). Similarly, administering treatment 6 hours after LPS challenge did not lead to significant alterations in BALF total protein levels or lung wet-to-dry ratios *in vivo* (Fig. S8A and B).

### Omadacycline’s effect on lung injury severity

The effects of omadacycline on the severity of LPS-induced acute lung injury were investigated, as assessed by histology and a semiquantitative histopathological scoring system. Lung pathology scoring incorporated factors such as the presence of neutrophils in alveolar and interstitial spaces, the formation of alveolar hyaline membranes, proteinaceous debris, and thickening of alveolar septa, allowing for comparison of severity across treatment groups.

**Fig 5:**
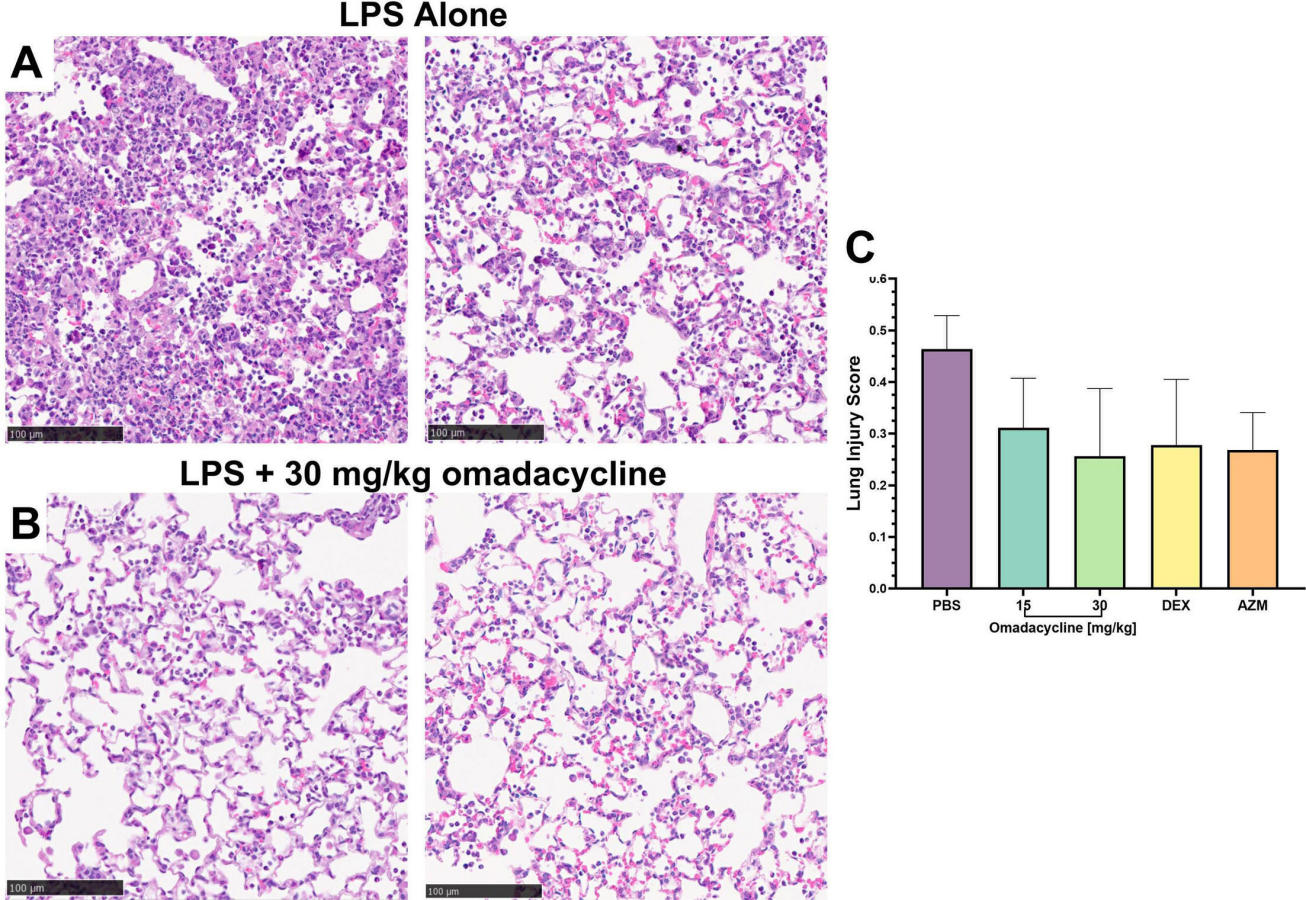
Dose effects of therapeutic omadacycline on acute lung injury severity. Representative photomicrographs of H&E-stained lung tissue for each (**A**) LPS alone and (B) LPS with 30 mg/kg omadacycline. (C) Evaluation of disease severity using a semiquantitative histopathological scoring system. Data are represented as mean and SEMs. *N* = 3 per treatment group. DEX = dexamethasone and AZM = azithromycin.

Treatment with omadacycline, dexamethasone, or azithromycin 1 hour prior to LPS challenge did not result in any noticeable improvements in ALI severity (Fig. S9). However, when these treatments were given 6 hours after LPS insufflation, a mild therapeutic effect was observed. A comparison of standardized lung injury scores demonstrated a reduction in lung injury in omadacycline-treated animals when compared to the control group. The effects of the 30 mg/kg omadacycline dose were comparable to those of dexamethasone (1 mg/kg IP) and azithromycin (30 mg/kg SC), indicating similar efficacy in mitigating lung injury (Fig. 5).

## DISCUSSION

In our analyses, we employed the well-established murine model of LPS-induced acute lung injury to simulate the host lung immune response and histopathological changes (27–29). Within these *in vivo* experiments, mice received omadacycline as both pre- and post-treatment for ALI to assess its preventive efficacy against ALI (pretreatment) and to mirror clinical scenarios involving the diagnosis of ALI followed by treatment initiation (post-treatment). Our *in vivo* findings have been substantiated by further *in vitro* investigations by our group. In the current investigation, we present the first evidence that omadacycline yields protective and therapeutic effects in mitigating ALI by reducing the production of proinflammatory cytokines and chemokines and neutrophil infiltration into the lungs, along with modestly improving lung injury severity.

Inflammation mediated by innate immunity plays a pivotal role in the pathophysiology of ALI. Following a direct insult, such as infection or trauma, cell-mediated increases in the production of proinflammatory cytokines occur in response to pathogens and ongoing cellular damage. Here, we used the well-established bacterial endotoxin model to simulate the host lung immune response. Upon intranasal LPS challenge, we found that omadacycline statistically significantly reduced the total airway exposure of several monocyte/macrophage-related cytokines (i.e., TNF-α, IL-1β, IL-6, CXCL-1, CXCL-2, and MMP-9). Notably, the suppression of pro-inflammatory cytokines was evident in both lung homogenates and BALF of mice treated with omadacycline. These findings underscore omadacycline’s ability to modulate cytokine levels across different treatment time points and sampling sites within the airway, highlighting its potential therapeutic value in mitigating inflammation in acute lung injury.

To investigate whether omadacycline directly targets macrophages, *in vitro* gene and protein expression experiments were performed using LPS-stimulated THP-1-derived macrophages. Gene expression results indicate that omadacycline downregulates the transcription of several pro-inflammatory cytokines and chemokines (IL-6, CXCL-1, and MMP-9). Subsequent protein expression experiments confirmed that treatment of omadacycline at higher doses inhibited the release of soluble TNF-α, IL-6, CXCL-1, and MMP-9 *in vitro*. These data are consistent with published literature highlighting the ability of omadacycline to modulate LPS-induced production of pro-inflammatory cytokines and chemokines, as well as suggest the macrophage as a primary cellular target of omadacycline (26).

A fundamental feature of acute lung injury is the vascular migration and interstitial migration of neutrophils into the lung compartment (39). In the murine model of ALI, we found that prophylactic omadacycline administration dose-dependently reduced leukocyte recruitment into the lungs *in vivo*. Additionally, neutrophil-to-macrophage ratios were also dose-dependently reduced across all omadacycline doses. Similar effects were also observed in mice in the dose-ranging ALI treatment study that received omadacycline treatment post-LPS insufflation. The significant decrease in WBC and neutrophil counts suggests that omadacycline may directly inhibit neutrophil migration from the vasculature. These data also support our prior observations, which indicate omadacycline’s capacity to suppress the release of CXCL-1 and CXCL-2, essential regulators involved in the migration and activation of neutrophils during murine LPS-induced pulmonary inflammation (40). To examine the direct impact of omadacycline on neutrophil chemotaxis, we conducted *in vitro* experiments using primary human neutrophils. Pretreatment with omadacycline significantly and dose-dependently inhibited human IL-8-dependent neutrophil chemotaxis. These findings suggest that omadacycline may also target peripheral blood neutrophils, thereby reducing the airway neutrophil burden through systemic action on this cell population.

We also investigated omadacycline’s ability to reduce the severity of lung injury and pulmonary edema *in vivo*. Normal lung tissue is characterized by thin alveolar walls with occasional intra-alveolar macrophages and very few neutrophils. Intranasal LPS challenge results in significant tissue injury within less than an hour, marked by neutrophil accumulation in the alveolar and interstitial spaces, thickening of the alveolar walls, and the presence of proteinaceous edema and debris in the alveolar space (38). Treatment with omadacycline 1 hour before the LPS challenge did not produce any noticeable improvements in ALI severity as assessed by a semiquantitative lung injury scoring system. However, administering the treatment 6 hours post-LPS insufflation showed a mild therapeutic effect. Prolonged and excessive neutrophil-driven inflammatory responses also contribute to basement membrane destruction and heightened permeability of the alveolar-capillary barrier. In the context of pulmonary edema, this heightened permeability disrupts the typical separation between the lung alveoli and capillaries, leading to the accumulation of fluid in the air spaces (38). The total protein content in BALF can serve as a marker of alveolar permeability and pulmonary edema. The severity of pulmonary edema was also assessed by measuring lung wet-to-dry ratios (38). However, omadacycline treatment did not demonstrate any significant reductions in pulmonary edema *in vivo*.

Pharmacokinetic studies play a pivotal role in identifying the optimal dosage for maximizing both the efficacy and safety of a compound. Pharmacokinetic analysis of omadacycline in mice was conducted to evaluate both plasma and lung tissue exposure. Noncompartmental analysis of plasma and BALF concentration-time curves revealed several significant observations. First, the plasma C_max_ and AUC_0-24_ values observed in our study were slightly lower than those reported in existing literature, likely due to the use of EDTA-coated tubes for plasma collection (34). Initial pharmacokinetic studies by Paratek Pharmaceuticals using EDTA-coated tubes revealed an underestimation of omadacycline levels, prompting a switch to heparin-coated tubes. The area under the BALF concentration-time curve also exceeded that of plasma. This is consistent with prior literature and pharmacokinetic studies on the bronchopulmonary distribution of omadacycline, which have demonstrated elevated drug concentrations in epithelial lining fluid and alveolar macrophages (18). Last, extensive pulmonary distribution likely accounts for the significant anti-inflammatory effects of omadacycline observed in this murine model of LPS-induced lung neutrophilia.

A comparison of the data derived from this investigation in mice indicates exposures that are comparable to human clinical data reported in the literature (18). In this study, four SC doses of omadacycline were administered to mice at 2.5, 7.5, 15, and 30 mg/kg. Allometric scaling, using an exponent of 0.67 for body surface area and assuming a 60 kg patient, indicated that these doses correspond to approximately 11, 32, 64, and 128 mg of IV omadacycline in humans (41). The highest dose of 30 mg/kg (equivalent to 128 mg IV in humans) yielded a plasma C_max_ of 0.85 ± 0.23 mg/L, a plasma AUC_0–24_ of 6.29 ± 0.49 mg*h/L, and a BALF AUC_0–24_ of 15.86 ± 2.87 mg*h/L (Table 1). For comparison, published human pharmacokinetic data for a 100 mg IV omadacycline report a plasma C_max_ of 2.12 ± 0.68 mg/L, a plasma AUC_0–24_ of 12.14 ± 3.22 mg*h/L, and an ELF AUC_0–24_ of 17.23 mg*h/L (18). Although our mouse-derived plasma C_max_ and AUC_0–24_ values are slightly reduced compared to those observed in humans, the BALF AUC_0–24_ aligns closely with the human ELF AUC_0–24_, suggesting comparability (18). Limitations in allometric scaling and interspecies variability, including differences in administration routes (SC in mice vs IV in humans), may contribute to these discrepancies. Additionally, the use of EDTA-coated tubes in plasma collection may have underestimated drug levels as discussed above. Overall, the trends in pharmacokinetic parameters suggest that the anti-inflammatory effects of omadacycline observed in mice at doses of 15–30 mg/kg may translate to humans receiving 100 mg IV or 300 mg orally (17). Furthermore, the observed anti-inflammatory effects at the lower dose of 7.5 mg/kg further support the likelihood of similar outcomes in humans, reinforcing the drug’s potential therapeutic benefits.

From investigations disclosed here, we postulate that multifunctional actions of omadacycline on early macrophage-driven inflammatory reactions and blood neutrophil emigration underlie the *in vivo* observations of reduced inflammation and neutrophil lung recruitment from LPS challenge. However, there are several limitations and areas worthy of future explorations worth mentioning. First, although LPS-induced ALI is a recommended model for testing potential therapeutics, it does not represent all ALI models. We did not explore the utility of omadacycline in the treatment of ALI induced by other causes (i.e., acid aspiration). Second, omadacycline treatment generally had a notably stronger impact on cytokine levels within lung tissue and only mild anti-inflammatory effects in the BALF and mild impacts on reducing the severity of lung injury. Ethical and practical considerations limited the number of mice we included, and statistical significance may have been achieved with more animals per group. While histopathological analysis and cytokine measurements in samples obtained through bronchoalveolar lavage exhibited some variability and did not reach statistical significance, the overall trends observed in the data complemented the findings from lung tissue analysis, providing valuable insights into the overall modulation of the inflammatory response by omadacycline treatment. Additionally, it should be noted that DMSO may have contributed synergistically to azithromycin’s effects, as recent research indicates that DMSO has its own immunomodulatory properties (42). Further studies investigating the potential mechanism of action through which omadacycline affects neutrophil chemotaxis would be informative. Neutrophil chemotaxis was significantly reduced *in vitro*, although the effective concentrations exceeded those observed *in vivo*. Although further research into the anti-inflammatory mechanism of omadacycline is needed, these findings indicate further clinical investigation is warranted.

### Conclusions

In conclusion, omadacycline exhibited potent anti-inflammatory effects *in vitro* and in a murine model of LPS-induced lung neutrophilia. These preclinical results suggest that omadacycline may provide dual anti-bacterial and anti-inflammatory activities relevant to the treatment of lung infections in PwCF and support additional clinical studies in these patients.

## SUPPLEMENTAL MATERIAL

Fig. S1 msphere.00671-24-s0001.tif Human neutrophil and THP-1 cell viability after omadacycline treatment.

Fig. S2 msphere.00671-24-s0002.tif Effects of omadacycline on mRNA expression levels of cytokines and chemokines from LPS-stimulated THP-1- derived macrophages.

Fig. S3 msphere.00671-24-s0003.tif Total and neutrophil cell counts in BALF after treatment of omadacycline and controls 6 hours after LPS challenge.

Fig. S4 msphere.00671-24-s0004.tif Dose effects of therapeutic omadacycline on lung homogenate cytokines and chemokines.

Fig. S5 msphere.00671-24-s0005.tif Dose effects of preventative omadacycline on BALF cytokines and chemokines.

Fig. S6 msphere.00671-24-s0006.tif Dose effects of therapeutic omadacycline on BALF cytokines and chemokines.

Fig. S7 msphere.00671-24-s0007.tif Dose effects of preventative omadacycline on total protein content in BALF and lung wet-to-dry ratios.

Fig. S8 msphere.00671-24-s0008.tif Dose effects of therapeutic omadacycline on total protein content in BALF and lung wet-to-dry ratios.

Fig. S9 msphere.00671-24-s0009.tif Dose effects of preventative omadacycline on acute lung injury severity using a semiquantitative histopathological scoring system.

Supplemental material msphere.00671-24-s0010.docx Supplemental figure captions and Table S1.

ASM does not own the copyrights to Supplemental Material that may be linked to, or accessed through, an article. The authors have granted ASM a non-exclusive, world-wide license to publish the Supplemental Material files. Please contact the corresponding author directly for reuse.
