# Supplementary figures and images for: Immunomodulatory activity of omadacycline in vitro and in a murine model of acute lung injury
Source: mSphere. 2024 Oct 30;9(11):e00671-24. doi: 10.1128/msphere.00671-24 (PMC11580420; doi:10.1128/msphere.00671-24)

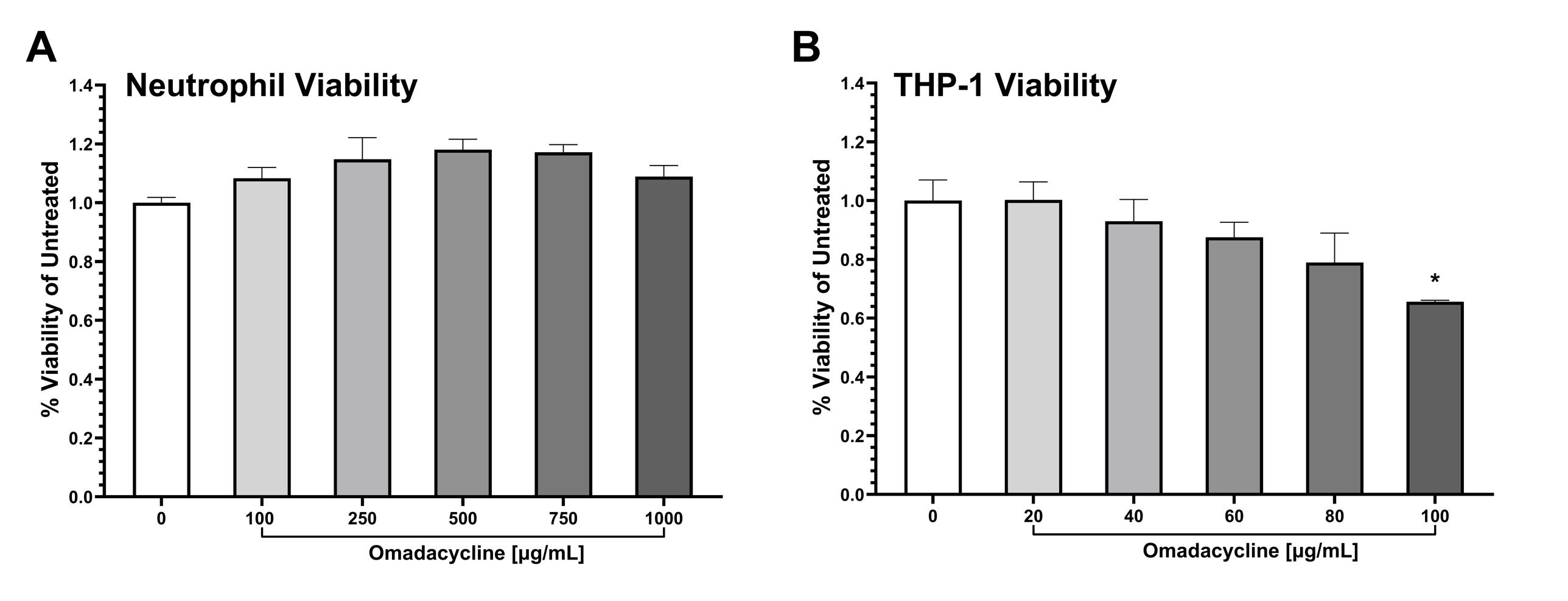

Supplement: Fig. S1 — Human neutrophil and THP-1 cell viability after omadacycline treatment. [file msphere.00671-24-s0001.tif]

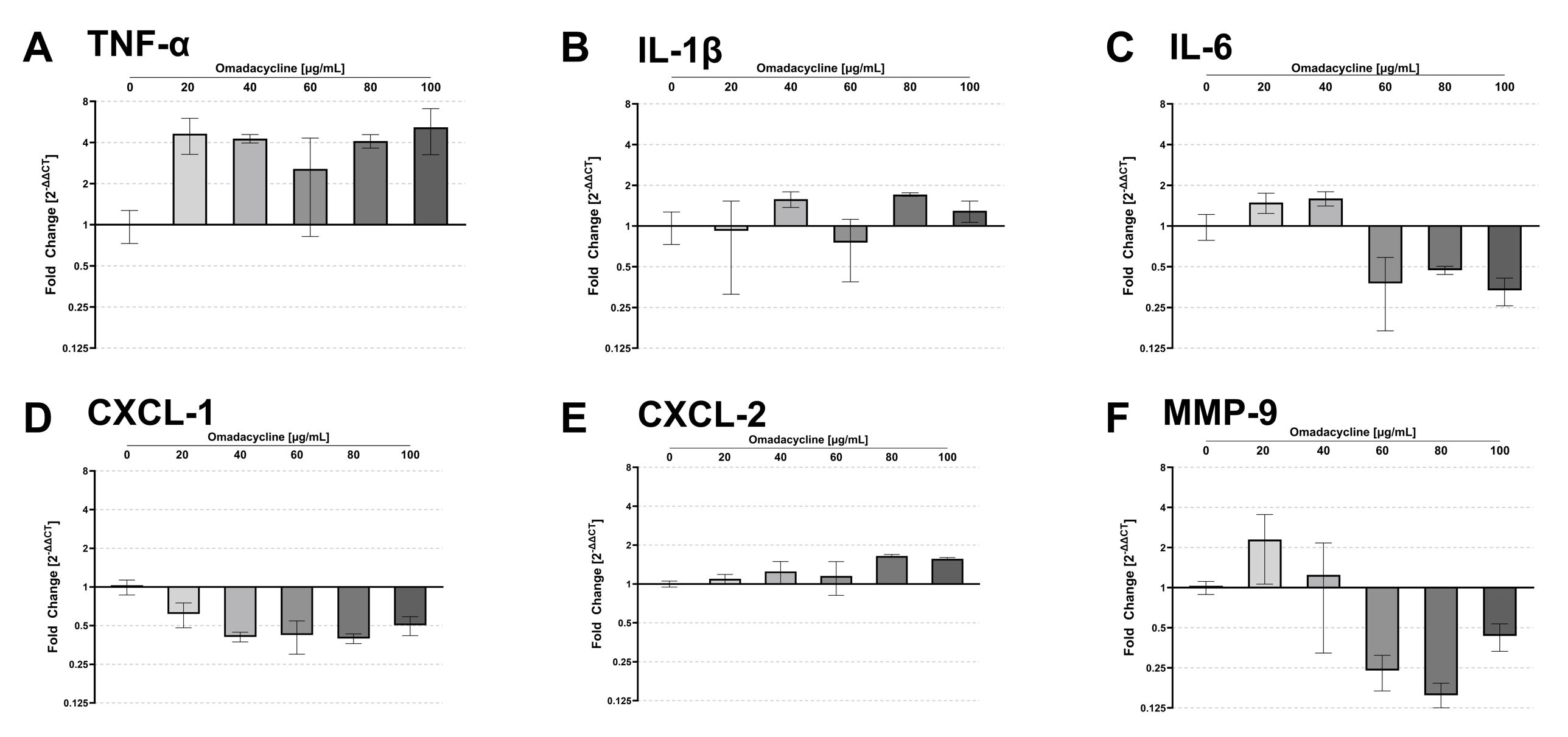

Supplement: Fig. S2 — Effects of omadacycline on mRNA expression levels of cytokines and chemokines from LPS-stimulated THP-1- derived macrophages. [file msphere.00671-24-s0002.tif]

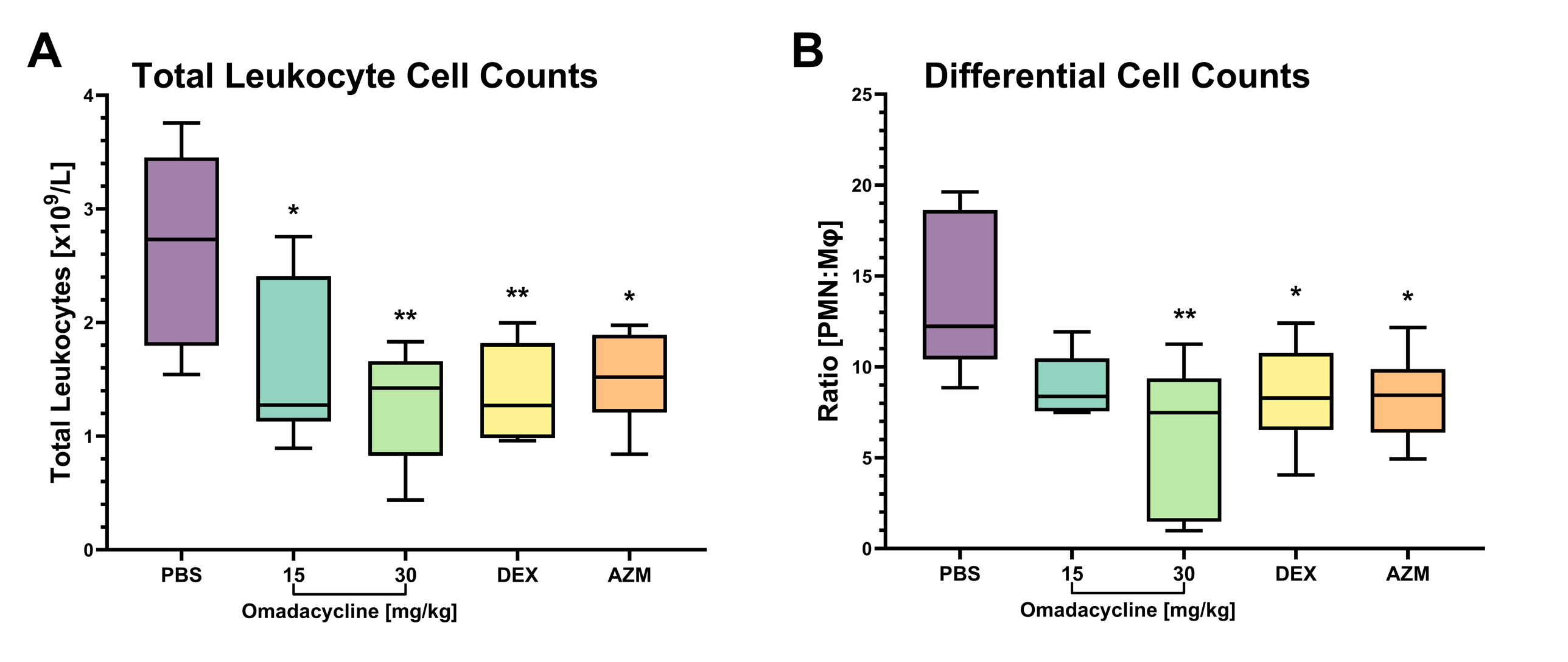

Supplement: Fig. S3 — Total and neutrophil cell counts in BALF after treatment of omadacycline and controls 6 hours after LPS challenge. [file msphere.00671-24-s0003.tif]

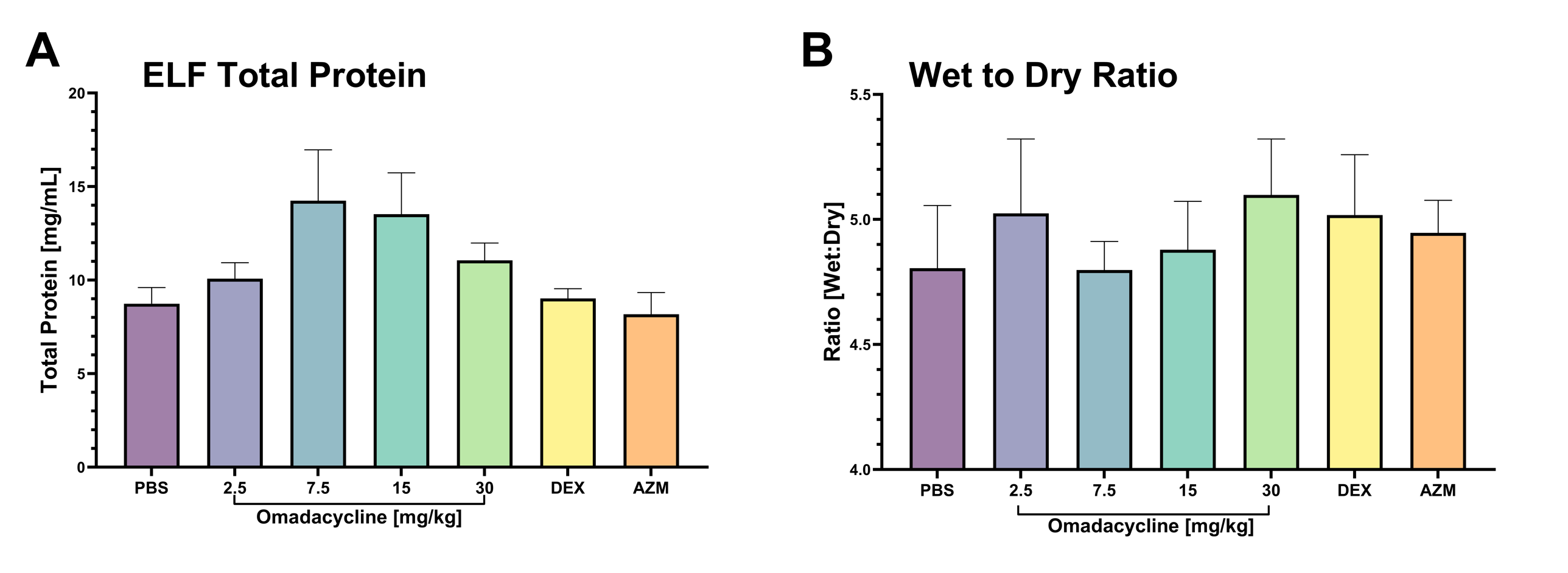

Supplement: Fig. S7 — Dose effects of preventative omadacycline on total protein content in BALF and lung wet-to-dry ratios. [file msphere.00671-24-s0007.tif]

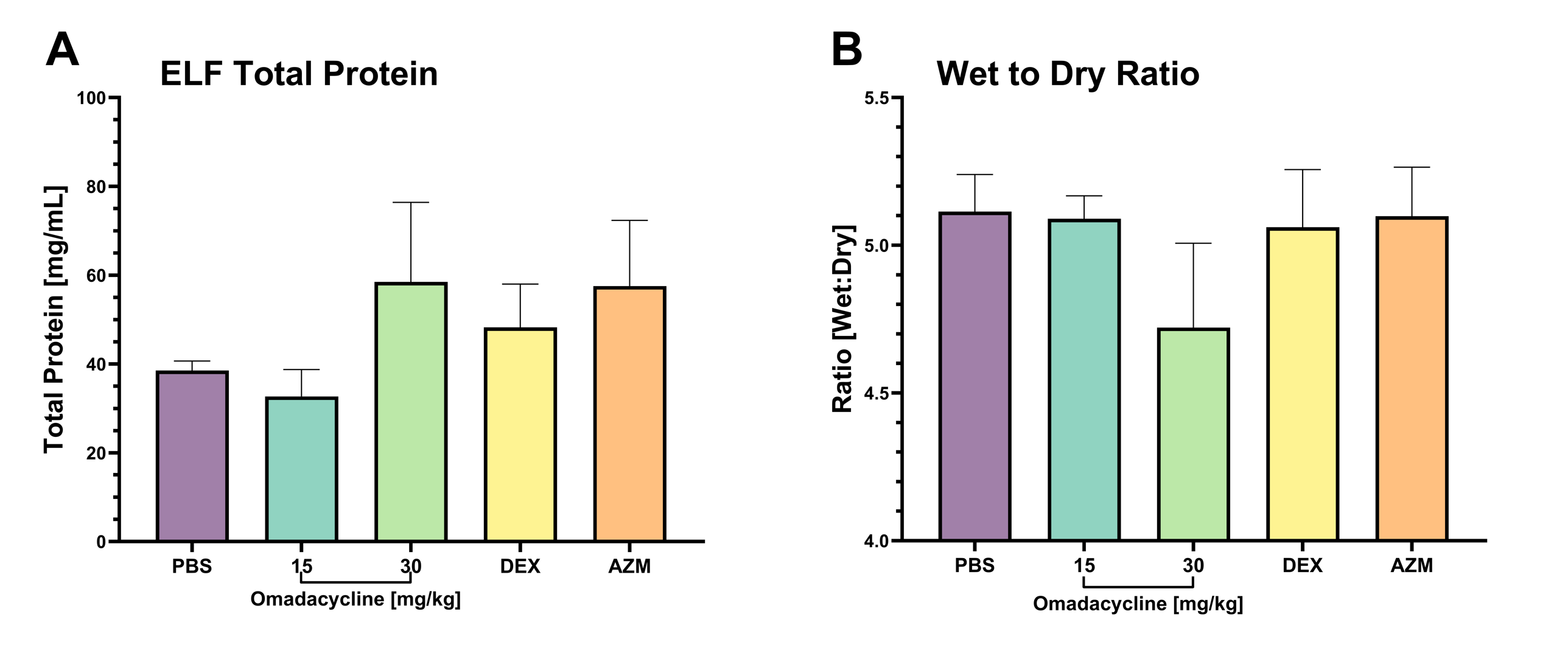

Supplement: Fig. S8 — Dose effects of therapeutic omadacycline on total protein content in BALF and lung wet-to-dry ratios. [file msphere.00671-24-s0008.tif]

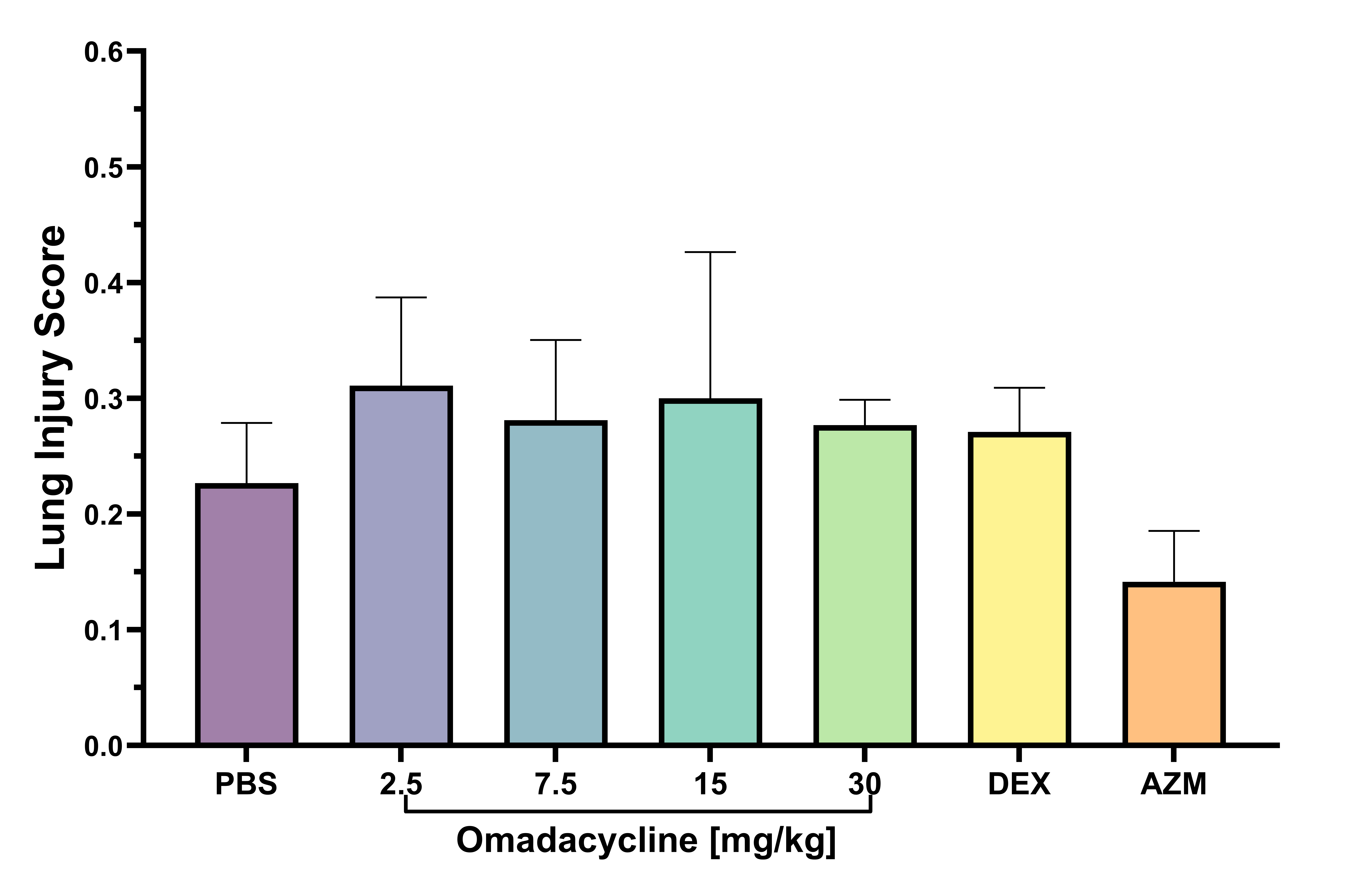

Supplement: Fig. S9 — Dose effects of preventative omadacycline on acute lung injury severity using a semiquantitative histopathological scoring system. [file msphere.00671-24-s0009.tif]
