# Supplementary material for: Immunomodulatory activity of omadacycline in vitro and in a murine model of acute lung injury
Source: mSphere. 2024 Oct 30;9(11):e00671-24. doi: 10.1128/msphere.00671-24 (PMC11580420; doi:10.1128/msphere.00671-24)
Supplement: Supplemental material — Supplemental figure captions and Table S1. [file msphere.00671-24-s0010.docx]

**Appendix:**

**Supplementary Figure S1:** (A) Human neutrophil viability after omadacycline treatment obtained by alamarBlue assay. (B) THP-1 cell viability after omadacycline treatment and LPS exposure obtained by alamarBlue assay. Cell viability was expressed as the percentage of control^^[[1]](#footnote-1)^^.

Data is represented as mean and standard errors of the mean. Asterisks indicate significant differences from vehicle-treated control cells.

(*P<0.05, post-hoc Tukey's HSD test)

**Supplementary Table S1:** Primers for human cytokines and housekeeping genes used for qualitative RT-PCR.

| **Gene** | **RefSeq** | **Gene name** | **Forward Primer (5′-3′)** | **Reverse Primer (5′-3′)** |
| --- | --- | --- | --- | --- |
| TNF-α | NM_000594.4 | Tumor necrosis factor-alpha | GCTGCACTTTGGAGTGATCG | GCTTGAGGGTTTGCTACAACA |
| IL-1β | NM_000576.3 | Interleukin-1 beta | GCTCGCCAGTGAAATGATGG | ATGGAGAACACCACTTGTTGC |
| IL-6 | NM_000600.5 | Interleukin-6 | GCCCACCGGGAACGAAAG | CGAAGGCGCTTGTGGAG |
| CXCL-1 | NM_001511.4 | Chemokine ligand 1 | CCCAAACCGAAGTCATAGCCA | AAGCCCCTTTGTTCTAAGCCA |
| CXCL-2 | NM_002089.4 | Chemokine ligand 2 | CAAACCGAAGTCATAGCCACA | TCGAAACCTCTCTGCTCTAACA |
| MMP-9 | NM_004994.3 | Matrix metalloproteinase-9 | CCTGGGCAGATTCCAAACCT | AACCGAGTTGGAACCACGAC |
| UBC | NM_021009.7 | Polyubiquitin-C | CAGCCGGGATTTGGGTCG | CACGAAGATCTGCATTGTCAAGT |

**Supplementary Figure S2:** Effects of omadacycline on mRNA expression levels of cytokines and chemokines from LPS-stimulated THP-1- derived macrophages. Gene expression was evaluated for (A) TNF-α, (B) IL-1β, (C) IL-6, (D) CXCL-1, (E) CXCL-2, (F) MMP-9.

Data is represented as mean and standard errors of the mean. The horizontal line represents a fold change of 1, indicating no difference in the transcription level compared to untreated control cells.

**Supplementary Figure S3:** (A) Total and (B) neutrophil cell counts in BALF after treatment of omadacycline and controls six hours after LPS challenge^^[[2]](#footnote-2)^^.

Box and whisker plots show 25% percentile, median, and 75% percentile in box, with minimum and maximum values shown with whiskers. Asterisks indicate significant differences from untreated control animals.

(*P<0.05, **P<0.01, post-hoc Tukey's HSD test)

PMN = polymorphonuclear neutrophil, Mφ= macrophage, DEX = dexamethasone, AZM = azithromycin

**Supplementary Figure S4:** Dose effects of therapeutic omadacycline on lung homogenate cytokines and chemokines. Lung homogenate supernatant was evaluated for (A) TNF-α, (B) IL-1β, (C) IL-6, (D) CXCL-1, (E) CXCL-2, (F) MMP-9^^[[3]](#footnote-3)^^.

Data is represented as mean and standard errors of the mean. Asterisks indicate significant differences from untreated control animals.

(*P<0.05, **P<0.01, post-hoc Tukey's HSD test)

DEX = dexamethasone, AZM = azithromycin

**Supplementary Figure S5:** Dose effects of preventative omadacycline on BALF cytokines and chemokines. BAL fluid was evaluated for (A) TNF-α, (B) IL-1β, (C) IL-6, (D) CXCL-1, (E) CXCL-2, (F) MMP-9.

Data is represented as mean and standard errors of the mean.

DEX = dexamethasone, AZM = azithromycin

**Supplementary Figure S6:** Dose effects of therapeutic omadacycline on BALF cytokines and chemokines. BAL fluid was evaluated for (A) TNF-α, (B) IL-1β, (C) IL-6, (D) CXCL-1, (E) CXCL-2, (F) MMP-9^^[[4]](#footnote-4)^^.

Data is represented as mean and standard errors of the mean.

DEX = dexamethasone, AZM = azithromycin

**Supplementary Figure S7:** Dose effects of preventative omadacycline on (A) total protein content in BALF and (B) lung wet-to-dry ratios.

Data is represented as mean and standard errors of the mean.

DEX = dexamethasone, AZM = azithromycin

**Supplementary Figure S8:** Dose effects of therapeutic omadacycline on (A) total protein content in BALF and (B) lung wet-to-dry ratios.

Data is represented as mean and standard errors of the mean.

DEX = dexamethasone, AZM = azithromycin

**Supplementary Figure S9:** Dose effects of preventative omadacycline on acute lung injury severity using a semiquantitative histopathological scoring system.

Data is represented as mean and standard errors of the mean. N=3 per treatment group.

DEX = dexamethasone, AZM = azithromycin

1. The alamarBlue assay was used to investigate the viability of THP-1-derived macrophages treated with omadacycline. As observed, treatment with omadacycline 30 min prior to LPS stimulation reduced THP-1-derived macrophage cell viability in a dose dependent manner. A statistically significant decrease in cell viability was observed at the highest omadacycline concentration (100 µg/mL) (P<0.05; Figure S1). [↑](#footnote-ref-1)
2. In the dose-ranging treatment study, administering omadacycline at doses of 15 and 30 mg/kg SC six hours after intranasal LPS challenge revealed a dose-dependent reduction in total WBC count and neutrophil recruitment to the lungs, observed in BAL measurements 48h post-challenge compared to the control group treated with PBS only. At both omadacycline doses, total WBC counts and neutrophil to macrophage ratios were significantly reduced compared with PBS control. These reductions were comparable to or more potent than dexamethasone (1 mg/kg IP) and azithromycin (30 mg/kg SC), with a tendency towards more potent inhibition of total cell counts and neutrophil recruitment at the higher 30 mg/kg omadacycline dose (Figure S3). [↑](#footnote-ref-2)
3. To examine the therapeutic effects of omadacycline on pro-inflammatory cytokines, chemokines, and MMP-9 induced by LPS, ELISA analysis was carried out on mouse lung homogenate (Figures S3*A-F*). Significant inhibition of TNF-α (P<0.05), IL-6 (P<0.01), and CXCL-2 (P<0.05) was observed at the 15 mg/kg omadacycline dose compared to PBS control (Figures S4*A*, S4*C*, and S4*F*). No treatment differences were observed in the levels of IL-1β, CXCL-1, or MMP-9 (Figures S4*B*, S4*D*, and S4*F*). [↑](#footnote-ref-3)
4. To assess the therapeutic effects of omadacycline on LPS-induced pro-inflammatory cytokines, chemokines, and MMP-9, BALF samples were evaluated by ELISA (Figures S4A-F). Despite the absence of statistically significant differences, treatment with 15 mg/kg omadacycline resulted in substantial reductions in TNF-α, IL-1β, IL-6, CXCL-1, CXCL2, and MMP-9 concentrations. These reductions were comparable to or more potent than dexamethasone (1 mg/kg IP) and azithromycin (30 mg/kg SC). However, no discernible treatment disparities in cytokine, chemokine, or MMP-9 levels were noted for the 30 mg/kg omadacycline doses (Figures S5*A-F*). [↑](#footnote-ref-4)
